# The ubiquitin-proteasome system in glioma cell cycle control

**DOI:** 10.1186/1747-1028-7-18

**Published:** 2012-07-20

**Authors:** Panagiotis J Vlachostergios, Ioannis A Voutsadakis, Christos N Papandreou

**Affiliations:** 1 Department of Medical Oncology, University Hospital of Larissa, University of Thessaly School of Medicine, Larissa, 41110, Greece; 2 Centre Pluridisciplinaire d’ Oncologie, Centre Hospitalier Universitaire Vaudois, Lausanne, Switzerland

**Keywords:** Ubiquitin-proteasome system, Glioma, Proteasome inhibitors, Ubiquitin, Cell cycle

## Abstract

A major determinant of cell fate is regulation of cell cycle. Tight regulation of this process is lost during the course of development and progression of various tumors. The ubiquitin-proteasome system (UPS) constitutes a universal protein degradation pathway, essential for the consistent recycling of a plethora of proteins with distinct structural and functional roles within the cell, including cell cycle regulation. High grade tumors, such as glioblastomas have an inherent potential of escaping cell cycle control mechanisms and are often refractory to conventional treatment. Here, we review the association of UPS with several UPS-targeted proteins and pathways involved in regulation of the cell cycle in malignant gliomas, and discuss the potential role of UPS inhibitors in reinstitution of cell cycle control.

## Introduction

Malignant gliomas constitute a spectrum of poorly differentiated primary brain tumors of astrocytic or oligodendroglial origin with a marked resistance to treatment, a high tendency of recurrence and a poor prognosis [1,2].

Deregulation of cell cycle in most cancer cell types, including glioma is a critical mechanism of development, progression, and resistance to treatment [3]. Aberrant function of critical regulators of the cell cycle generally results in modification of growth, differentiation and apoptotic properties of the cell. Knowledge of the regulatory mechanisms that govern the function of cell cycle controllers is critical to enable design of new or use of already existing inhibitors with the aim of inducing a cell cycle related anticancer effect.

The ubiquitin-proteasome system (UPS) is long-known as a cellular tool for the marking and proteolytic degradation of proteins involved in a wide variety of structural and functional roles inside the cell. The UPS includes the “ubiquitously” expressed 76-amino acid protein ubiquitin (Ub), the multisubunit protein organelle 26S proteasome, consisting of one 20S catalytic and two 19S regulatoty subunits, and finally, a 3-step enzymatic cascade of Ub-activating (E1), Ub-conjugating (E2) and Ub-ligase (E3) enzymes which attach ubiquitin to the target protein [4,5].

In cancer, a great number of cellular proteins with various roles, including cell cycle control, either comprise direct targets of an aberrant degradation machinery or have a close structural or/and functional connection with abnormal ubiquitin- or ubiquitin like-ligases, deubiquitinating enzymes and UPS-regulated signaling factors and pathways [6-8]. In this context, the involvement of UPS in cell cycle regulation is critical.

## Ubiquitination and proteasomal degradation of glioma cell cycle proteins

The earliest evidence indicative of an association between the stability of cell cycle proteins and UPS in gliomas concerns the cyclin-dependent kinase inhibitor p27, which was proved to be degraded in a proteasome-dependent manner [9,10]. p27 is a negative regulator of cyclin D-Cdk4, cyclin E-Cdk2 and cyclin A-Cdk2, being involved in G1-S transition, and its expression was observed to decrease with advancing anaplasia of astrocytic tumors. This feature was correlated with and, at least partially, attributed to increased levels of degradation activity [9]. This finding is further supported by evidence of an inverse correlation between levels of p27 and Skp2 (S-phase kinase-associated protein 2), a member of the ubiquitin ligase F-box family of proteins that promotes G1-S transition through targeting of p27 for degradation. p27 decreased with anaplasia and almost disappeared in glioblastomas (GBMs), whereas Skp2 was absent or poorly expressed in well-differentiated astrocytomas and it was diffusely or focally expressed in most GBMs [11].

p21 is another cell cycle-related protein target of the ubiquitin-proteasome pathway in gliomas. Being an established Cdk2 inhibitor (and likely Cdk1 and Cdk3 as well), p21 was found to be dependent on the ubiquitin ligase APC/C^Cdc20^ (Anaphase Promoting Complex/Cyclosome and its activator Cdc20) for its proteolytic degradation by the proteasome in prometaphase [12]. The suggested model involves a positive feedback loop where Cdk1, by phosphorylating certain subunits of APC/C^Cdc20^, promotes the activity of APC/C^Cdc20^, consequently triggering the degradation of p21, resulting in a further activation of Cdk1. The degradation of p21 via APC/C^Cdc20^ contributes to the full activation of Cdk1 in early M phase and prevents mitotic slippage during activation of the spindle assembly checkpoint [12]. The APC/C^Cdc20^ regulates the UPS-dependent degradation of several proteins that drive the cell cycle, including cyclins A and B, while other APC/C^Cdc20^ substrates (geminin, survivin, polo and aurora kinases) or regulators (Emi1, RASSF1A) are overexpressed in high grade gliomas [13]. p21 is also a transcriptional target of p53 and a negative regulator of the proteasome-dependent stability of p53- and Rb [14,15] whose intimate associations with the UPS are well-established and analyzed below.

The migration and invasion inhibitor protein (MIIP, also known as IIp45) was discovered as a negative regulator of cell migration and invasion in glioma and its expression was reduced or undetectable in tissue samples obtained from patients with GBM. At the cellular level, an important role was revealed for MIIP in the inhibition of gliomagenesis and attenuation of mitotic transition based on its association with APC/C activity. MIIP interacts directly with Cdc20, and this interaction of inhibits APC/C-mediated degradation of cyclin B1, thereby causing attenuation of mitotic transition and increased mitotic catastrophe [16]. Thus, loss of MIIP promotes cell cycle progression and mitotic activity during development and progression of gliomas.

Cdk1 (also named Cdc2) and cyclin B1 are also targets of the proteasome as evidenced from a study using geldanamycin, a drug interfering with the function of the chaperone protein Hsp90 (Heat shock protein 90), in glioma cells. Geldamycin promotes proteasome degradation of client proteins by inhibiting Hsp90 function [17]. In addition to Cdk1, proteasome-mediated degradation of kinase chk1 (check point kinase 1), a Cdk1 regulator, is also promoted by geldanamycin [18].

The cyclin A/Cdk2 complex was also demonstrated to be a proteasome substrate after being tethered to a constructed chimeric protein composed of an F-box protein (TrCP) fused to a cyclin A/Cdc2 inhibitor binding peptide. Moreover, this proteasome-mediated destruction resulted in massive tumor cell (including glioma) apoptosis both *in vitro* and *in vivo*. The explanation provided, was based on unopposed E2F1 (a cell cycle regulatory transcription factor) activity which is further enhanced in Rb-deregulated cells [19].

UPS is also involved in the regulation of cyclin D1 protein levels. This is effected through its phosphorylation by the IKKα (Inhibitor of kappa B kinase alpha), which in turn drives the protein from nucleus to the cytoplasm, targeting it for ubiquitination and subsequent proteolytic degradation by the proteasome [20]. Of note is that this translocation preceding proteolytic degradation is favoured by acidic environment [21]. On the other hand, UPS is also “present” in cyclin D1 upregulation via the UPS-regulated transcription factor NF-κB, activation of which contributes to cell cycle progression and prevents differentiating GBM-initiating cells (GICs) from acquiring a mature postmitotic phenotype [22].

The E2F1 protein, a cellular mediator involved in the transition from G1 to S phase was found to be degraded by the proteasome in response to Δ9-tetrahydrocannabinol treatment of human GBM cells, thus blocking cell cycle progression and supporting a role for cannabinoids as anticancer agents [23]. The first report implicating the UPS in E2F1 regulation had revealed a degradation target sequence in a carboxyl-terminal region of E2F1 as well as a stabilizing role for Rb tumor suppressor protein in this process [24].

A relatively new cell cycle regulatory protein in malignant astrocytoma, Pescadillo, is subjected to post-translational modification by SUMO-1 (small ubiquitin-like modifier, a member of the ubiquitin protein family). Pescadillo protein expression is upregulated after loss of p53, contributing to cell cycle progression and proliferation. This SUMO-1 modification may be important for protein targeting and protein-protein interactions, although it is probably not involved in a proteolytic control of the protein-conjugate, as derived from data obtained from yeast and human cells, in which SUMO-1 conjugates remained unaffected by proteasome inhibition [25].

There are also several implications for an indirect role of UPS in cell cycle, mediated through the regulation of stability of the oncoprotein c-Myc, which is a known activator of cell cycle acceleration. Interestingly, the DNA-dependent protein kinase catalytic subunit (DNA-PKcs) has emerged as a novel modulator of this ubiquitin-mediated c-Myc proteolysis via Akt /glycogen synthase kinase 3 (GSK3) pro-survival pathway in human glioma cells [26]. The importance of c-Myc downregulation in relation with cell cycle responses in astrocytoma cell lines involves a G1 arrest together with inhibition of DNA synthesis and delay in S phase transition. These effects emanate from loss of cyclin E and cyclin A proteins accompanied by inhibition of the cyclin D1/Cdk4 complex activity (as evaluated by presence of its active unphosphorylated target substrate, Rb) via p21 and p57 assembly [27]. Notably, Rb, which plays a key role in regulation of cell cycle, primarily via interaction with the E2F family of transcription factors in assembly of active repressor complexes to negatively regulate expression of E2F-dependent genes important for cell cycle progression, is itself a proteasome target protein. This regulation of Rb protein levels is governed by an Mdm2-mediated promotion of Rb-20S proteasome subunit interaction leading to a proteasome-dependent ubiquitin-independent degradation of Rb [28].

Mutations of the gene PARK2, which encodes an E3 ubiquitin ligase, parkin, is common not only in early-onset familial Parkinson’s disease but also in cancer, as they occur in the same domains, and sometimes at the same residues. The significance of PARK2 mutations in various cancers, including GBM, lies in the fact that the subsequent decrease in PARK2’s E3 ligase activity compromises its ability to ubiquitinate cyclin E and thereby results in mitotic instability [29,30]. Parkin expression is dramatically reduced in glioma cells. Restoration of parkin expression promotes G1 phase cell cycle arrest and attenuates the proliferation rate of glioma cells *in vitro* and *in vivo*. Notably, parkin-expressing glioma cells have reduced levels of cyclin D1, but not cyclin E, and a selective downregulation of Akt serine-473 phosphorylation and VEGF receptor levels. Reversely, parkin-null mouse models exhibit increased levels of cyclin D1, VEGF receptor, and Akt phosphorylation, and divide significantly faster when compared with wild-type cells, with suppression of these changes following parkin reintroduction. At the clinical level, a prognostic role of the parkin pathway has emerged from studying the survival outcome of patients with glioma according to parkin status. Parkin expression in GBM patients is associated with lower grade and improved survival [31].

Regulator of Cullins-1 (ROC1) or Ring Box Protein-1 (RBX1) is a RING component of SCF (Skp-1, cullins, F-box proteins) E3 ubiquitin ligases implicated in cell growth and cell cycle progression of GBM. The latter was shown to be attenuated after ROC1 silencing by siRNA which caused growth inhibition, induction of senescence, apoptosis and G2/M arrest in U87 GBM cells. Senescence induction was coupled with DNA damage in p53/p21- and p16/Rb-independent manners. Apoptosis was associated with accumulation of Puma (p53 upregulated modulator of apoptosis) and reduction of Bcl-2, Mcl-1, (Myeloid cell leukemia sequence 1) and survivin, and G2/M arrest was associated with accumulation of 14-3-3sigma and elimination of cyclin B1 and Cdc2 [32].

Last but not least, the induction of G0/G1 or G2/M cell cycle arrest and/or apoptosis, whenever indicated as a response to various stressors, could not be ensured without the critical involvement of p53, whose levels and activation within the ARF-Mdm2-p53 axis are under tight UPS-dependent regulation. Phosphorylation in response to genotoxic stimuli, acetylation and deubiquitination enhance p53 stabilization and activation via attenuated interaction with Mdm2 and inhibition or reversal of ubiquitination. Phosphorylation of the E3-ligase Mdm2 may either suspend nuclear export of p53 or promote nuclear translocation of Mdm2 with adverse consequences on p53 stability, depending on the inducing kinase (ATM signaling stabilizes p53, Akt signalling destabilizes it). ARF can induce the accumulation of p53 by repressing Mdm2, and all three members of the axis (ARF, Mdm2, p53) exhibit non-overlapping activities and participate in autoregulatory feedback loops involving p53-Mdm2 and p53-ARF doublets [33]. The p53 pathway plays a crucial role in the development of secondary GBMs, thought to be derived to the malignant progression to grade II or III astrocytomas as p53 mutations are rather common (65 %) in this subtype [34,35]. Amplification of Mdm2 is rare but amplification and overexpression of MdmX, which is a p53-binding protein with close homology to Mdm2, has been found in certain malignant gliomas that have wild-type p53 [36]. Further, the alternative splicing of Mdm2 has been correlated with stabilized wild-type p53 in certain human GBM cells [37]. Newer studies have ascribed a role for merlin, a neurofibromatosis 2 (NF2)-related tumor suppressor in promotion of p53 stability and activity by inducing Mdm2 degradation in glioma cells [38]. p53 protection from Mdm2-mediated degradation is also conferred by PTEN via inhibition of PI3K/Akt signaling that promotes Mdm2 nuclear translocation. Furthermore, activated p53 induces PTEN gene expression, providing evidence for a positive feedback loop, amplifying sensitization of glioma cells to chemotherapy that relies on p53 activity [39]. The process of post-translational p53 stabilization further involves reversible protein phosphorylation by a kinase/phosphatase pair. In general, phosphorylation of p53 blocks the p53-Mdm2 interaction and thus stabilizes p53 in stressed cells. A newly identified complex GAS41-PP2Cβ is specifically required for dephosphorylation of serine 366 on p53 and this is highly relevant in human glioma given that GAS41 is frequently amplified in this type of malignancy [40]. Thus, p53 regulation by UPS in malignant gliomas is a promising target, in terms of proteasome inhibition, or ubiquitination-resistant p53 protein transduction therapy [41]. A stable mutant p53 in which all lysines were replaced by arginines making it ubiquitination-resistant was found to be transcriptionally active and to inhibit glioma cell proliferation. If transduction efficacy could be ensured *in vivo*, a stable ubiquitination-resistant p53 could become a therapeutically useful intervention in malignant gliomas.

**Figure 1:**
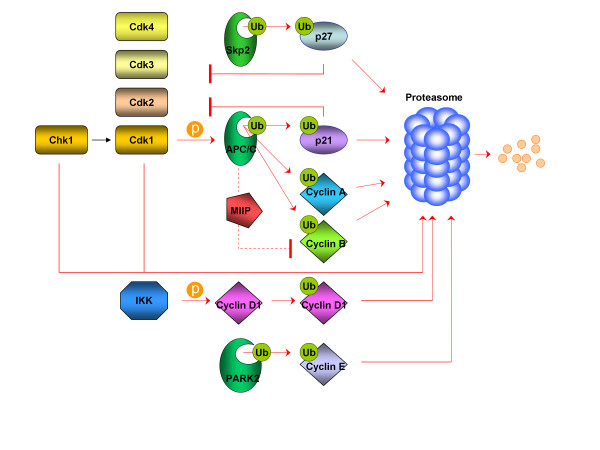
UPS-dependent regulation of cyclins, Cdks and Cdk inhibitors in GBM.

**Figure 2:**
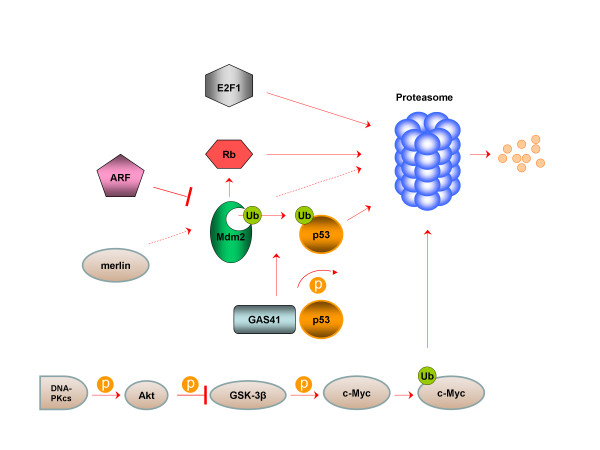
UPS-dependent regulation of cell cycle-associated transcription factors in GBM.

Most important cell cycle regulators that are intimately associated with the UPS, either members of the basal cell cycle machinery (cyclins, Cdks, Cdk inhibitors) or cell cycle-related transription factors are depicted in Figures 1 and 2, respectively.

## The role of proteasome inhibition in glioma cell cycle

The regulation of turnover of many cell cycle effectors by the UPS in gliomas has led to the consideration of proteasome inhibition as a potential modulator of cell cycle abnormalities in these tumors. Overall, an illustrative picture of the changes in cell cycle of GBM cell lines, induced by a proteasome inhibitor, includes a G2/M cell cycle arrest with a concomitant decreased percentage of cells in S phase. This is associated with an increased expression of p21, p27, cyclin D1 and decreased levels of Cdk2, Cdk4 and E2F4 (member of the E2F family of transcription factors) [42]. As data from basic and preclinical research continue to accumulate, existing information on the role of UPS in glioma cell cycle regulation is steadily confirmed by the use of novel proteasome inhibitors, such as SC68896 and celastrol [43,44]. Accumulation of G2/M phase-related proteins p21 and p27 was the most prominent cell cycle-related event after treatment with SC68896. This was accompanied by caspase cleavage, and sensitization of glioma cells to TRAIL and CD95L via Upregulation of cell surface expression of the death receptors DR4 and DR5 [43]. Upregulation of p21, p27 and cyclin B1 together with downregulation of Cdk2 were observed with celastrol. Cell cycle arrest was followed by proapoptotic Bax and caspase-3 stabilization as well as reduced levels of antiapoptotic Bcl-2 and XIAP proteins [44]. In addition, a new role has been revealed for the gamma-secretase inhibitor LLNIe as it was shown to cause G2/M arrest and subsequent apoptosis in cells with GBM tumor-initiating cells (TICs) phenotype by inducing proteasome inhibition and proteolytic stress [45]. High concentrations of ritonavir, an anti-retroviral medication recently found to be a proteasome inhibitor, induced a cell cycle arrest in the G1 phase followed by apoptosis of GL15 glioma cells [46].

## Conclusions

This review was intended to offer an insight to the role of the UPS in regulation of ubiquitination and stability of several cell cycle proteins rather than cover all aspects of cell cycle events being under the control of UPS in malignant gliomas, as well as of the complexity and extent of this control. The use of proteasome inhibition as an anticancer treatment is possibly meaningful in terms of cell cycle control, and combinations with other agents with established activity against aberrant cell cycle progression or under investigation might enable a more efficient strategy for rapidly proliferating tumors such as malignant gliomas.

## Competing interests

The authors declare that they have no competing interests.

## Authors’ contributions

PJV performed literature review and drafted the manuscript. IAV and CNP revised and approved the final manuscript. All authors read and approved the final manuscript.

## References

[B1] Preusser M Haberler C Hainfellner JA Malignant glioma: neuropathology and neurobiology Wien Med Wochenschr 2006 156 332 337 10.1007/s10354-006-0304-7 16944363

[B2] Wen PY Kesari S Malignant gliomas in adults N Engl J Med 2008 359 492 507 10.1056/NEJMra0708126 18669428

[B3] Hanahan D Weinberg RA Hallmarks of cancer: the next generation Cell 2011 144 646 674 10.1016/j.cell.2011.02.013 21376230

[B4] Ciechanover A Orian A Schwartz AL Ubiquitin-mediated proteolysis: biological regulation via destruction Bioessays 2000 22 442 451 10.1002/(SICI)1521-1878(200005)22:5<442::AID-BIES6>3.0.CO;2-Q 10797484

[B5] Adams J The proteasome: structure, function, and role in the cell Cancer Treat Rev 2003 29 Suppl 1 3 9 12738238 10.1016/s0305-7372(03)00081-1

[B6] Mani A Gelmann EP The ubiquitin-proteasome pathway and its role in cancer J Clin Oncol 2005 23 4776 4789 10.1200/JCO.2005.05.081 16034054

[B7] Burger AM Seth AK The ubiquitin-mediated protein degradation pathway in cancer: therapeutic implications Eur J Cancer 2004 40 2217 2229 10.1016/j.ejca.2004.07.006 15454246

[B8] Hoeller D Hecker CM Dikic I Ubiquitin and ubiquitin-like proteins in cancer pathogenesis Nat Rev Cancer 2006 6 776 788 10.1038/nrc1994 16990855

[B9] Piva R Cancelli I Cavalla P Bortolotto S Dominguez J Draetta GF Schiffer D Proteasome-dependent degradation of p27/kip1 in gliomas J Neuropathol Exp Neurol 1999 58 691 696 10.1097/00005072-199907000-00002 10411338

[B10] Pamarthy D Tan M Wu M Chen J Yang D Wang S Zhang H Sun Y p27 degradation by an ellipticinium series of compound via ubiquitin-proteasome pathway Cancer Biol Ther 2007 6 360 366 10.4161/cbt.6.3.3703 17312389

[B11] Schiffer D Cavalla P Fiano V Ghimenti C Piva R Inverse relationship between p27/Kip.1 and the F-box protein Skp2 in human astrocytic gliomas by immunohistochemistry and Western blot Neurosci Lett 2002 328 125 128 10.1016/S0304-3940(02)00483-4 12133571

[B12] Amador V Ge S Santamaría PG Guardavaccaro D Pagano M APC/C(Cdc20) controls the ubiquitin-mediated degradation of p21 in prometaphase Mol Cell 2007 27 462 473 10.1016/j.molcel.2007.06.013 17679094 PMC2000825

[B13] Lehman NL Tibshirani R Hsu JY Natkunam Y Harris BT West RB Masek MA Montgomery K van de Rijn M Jackson PK Oncogenic regulators and substrates of the anaphase promoting complex/cyclosome are frequently overexpressed in malignant tumors Am J Pathol 2007 170 1793 1805 10.2353/ajpath.2007.060767 17456782 PMC1854971

[B14] Broude EV Demidenko ZN Vivo C Swift ME Davis BM Blagosklonny MV Roninson IB p21 (CDKN1A) is a negative regulator of p53 stability Cell Cycle 2007 6 1468 1471 17585201

[B15] Broude EV Swift ME Vivo C Chang BD Davis BM Kalurupalle S Blagosklonny MV Roninson IB p21(Waf1/Cip1/Sdi1) mediates retinoblastoma protein degradation Oncogene 2007 26 6954 6958 10.1038/sj.onc.1210516 17486059

[B16] Ji P Smith SM Wang Y Jiang R Song SW Li B Sawaya R Bruner JM Kuang J Yu H Fuller GN Zhang W Inhibition of gliomagenesis and attenuation of mitotic transition by MIIP Oncogene 2010 29 3501 3508 10.1038/onc.2010.114 20418911

[B17] Nomura N Nomura M Newcomb EW Zagzag D Geldanamycin induces G2 arrest in U87MG glioblastoma cells through downregulation of Cdc2 and cyclin B1 Biochem Pharmacol 2007 73 1528 1536 10.1016/j.bcp.2007.01.022 17324379

[B18] Nomura M Nomura N Yamashita J Geldanamycin-induced degradation of Chk1 is mediated by proteasome Biochem Biophys Res Commun 2005 335 900 905 10.1016/j.bbrc.2005.07.160 16099423

[B19] Chen W Lee J Cho SY Fine HA Proteasome-mediated destruction of the cyclin a/cyclin-dependent kinase 2 complex suppresses tumor cell growth in vitro and in vivo Cancer Res 2004 64 3949 3957 10.1158/0008-5472.CAN-03-3906 15173007

[B20] Kwak YT Li R Becerra CR Tripathy D Frenkel EP Verma UN IkappaB kinase alpha regulates subcellular distribution and turnover of cyclin D1 by phosphorylation J Biol Chem 2005 280 33945 33952 10.1074/jbc.M506206200 16103118

[B21] Schnier JB Nishi K Harley WR Gorin FA An acidic environment changes cyclin D1 localization and alters colony forming ability in gliomas J Neurooncol 2008 89 19 26 10.1007/s11060-008-9591-8 18404250 PMC2996278

[B22] Nogueira L Ruiz-Ontañon P Vazquez-Barquero A Lafarga M Berciano MT Aldaz B Grande L Casafont I Segura V Robles EF Suarez D Garcia LF Martinez-Climent JA Fernandez-Luna JL Blockade of the NFκB pathway drives differentiating glioblastoma-initiating cells into senescence both in vitro and in vivo Oncogene 2011 30 3537 3548 10.1038/onc.2011.74 21423202

[B23] Galanti G Fisher T Kventsel I Shoham J Gallily R Mechoulam R Lavie G Amariglio N Rechavi G Toren A Delta 9-tetrahydrocannabinol inhibits cell cycle progression by downregulation of E2F1 in human glioblastoma multiforme cells Acta Oncol 2008 47 1062 1070 10.1080/02841860701678787 17934890

[B24] Campanero MR Flemington EK Regulation of E2F through ubiquitin-proteasome-dependent degradation: stabilization by the pRB tumor suppressor protein Proc Natl Acad Sci U S A 1997 94 2221 2226 10.1073/pnas.94.6.2221 9122175 PMC20068

[B25] Kinoshita Y Jarell AD Flaman JM Foltz G Schuster J Sopher BL Irvin DK Kanning K Kornblum HI Nelson PS Hieter P Morrison RS Pescadillo, a novel cell cycle regulatory protein abnormally expressed in malignant cells J Biol Chem 2001 276 6656 6665 10.1074/jbc.M008536200 11071894

[B26] An J Yang DY Xu QZ Zhang SM Huo YY Shang ZF Wang Y Wu DC Zhou PK DNA-dependent protein kinase catalytic subunit modulates the stability of c-Myc oncoprotein Mol Cancer 2008 7 32 10.1186/1476-4598-7-32 18426604 PMC2383926

[B27] Amendola D De Salvo M Marchese R Verga Falzacappa C Stigliano A Carico E Brunetti E Moscarini M Bucci B Myc down-regulation affects cyclin D1/cdk4 activity and induces apoptosis via Smac/Diablo pathway in an astrocytoma cell line Cell Prolif 2009 42 94 109 10.1111/j.1365-2184.2008.00576.x 19143767 PMC6496913

[B28] Ying H Xiao ZX Targeting retinoblastoma protein for degradation by proteasomes Cell Cycle 2006 5 506 508 10.4161/cc.5.5.2515 16552188

[B29] Veeriah S Taylor BS Meng S Fang F Yilmaz E Vivanco I Janakiraman M Schultz N Hanrahan AJ Pao W Ladanyi M Sander C Heguy A Holland EC Paty PB Mischel PS Liau L Cloughesy TF Mellinghoff IK Solit DB Chan TA Somatic mutations of the Parkinson’s disease-associated gene PARK2 in glioblastoma and other human malignancies Nat Genet 2010 42 77 82 10.1038/ng.491 19946270 PMC4002225

[B30] Veeriah S Morris L Solit D Chan TA The familial Parkinson disease gene PARK2 is a multisite tumor suppressor on chromosome 6q25.2–27 that regulates cyclin E Cell Cycle 2010 9 1451 1452 10.4161/cc.9.8.11583 20372088 PMC2921461

[B31] Yeo CW Ng FS Chai C Tan JM Koh GR Chong YK Koh LW Foong CS Sandanaraj E Holbrook JD Ang BT Takahashi R Tang C Lim KL Parkin pathway activation mitigates glioma cell proliferation and predicts patient survival Cancer Res 2012 72 2543 53 10.1158/0008-5472.CAN-11-3060 22431710

[B32] Jia L Soengas MS Sun Y ROC1/RBX1 E3 ubiquitin ligase silencing suppresses tumor cell growth via sequential induction of G2-M arrest, apoptosis, and senescence Cancer Res 2009 69 4974 4982 10.1158/0008-5472.CAN-08-4671 19509229 PMC2744327

[B33] Yang Y Li CC Weissman AM Regulating the p53 system through ubiquitination Oncogene 2004 23 2096 2106 10.1038/sj.onc.1207411 15021897

[B34] Steinbach JP Weller M Apoptosis in gliomas: molecular mechanisms and therapeutic implications J Neurooncol 2004 70 245 254 10.1007/s11060-004-2753-4 15674481

[B35] Ohgaki H Kleihues P Genetic pathways to primary and secondary glioblastoma Am J Pathol 2007 170 1445 1453 10.2353/ajpath.2007.070011 17456751 PMC1854940

[B36] Riemenschneider MJ Büschges R Wolter M Reifenberger J Boström J Kraus JA Schlegel U Reifenberger G Amplification and overexpression of the MDM4 (MDMX) gene from 1q32 in a subset of malignant gliomas without TP53 mutation or MDM2 amplification Cancer Res 1999 59 6091 6096 10626796

[B37] Kraus A Neff F Behn M Schuermann M Muenkel K Schlegel J Expression of alternatively spliced mdm2 transcripts correlates with stabilized wild-type p53 protein in human glioblastoma cells Int J Cancer 1999 80 930 934 10.1002/(SICI)1097-0215(19990315)80:6<930::AID-IJC20>3.0.CO;2-M 10074928

[B38] Kim H Kwak NJ Lee JY Choi BH Lim Y Ko YJ Kim YH Huh PW Lee KH Rha HK Wang YP Merlin neutralizes the inhibitory effect of Mdm2 on p53 J Biol Chem 2004 279 7812 7818 14679203 10.1074/jbc.M305526200

[B39] Mayo LD Donner DB The PTEN, Mdm2, p53 tumor suppressor-oncoprotein network Trends Biochem Sci 2002 27 462 467 10.1016/S0968-0004(02)02166-7 12217521

[B40] Park JH Smith RJ Shieh SY Roeder RG The GAS41-PP2Cbeta complex dephosphorylates p53 at serine 366 and regulates its stability J Biol Chem 2011 286 10911 10917 10.1074/jbc.C110.210211 21317290 PMC3064146

[B41] Michiue H Tomizawa K Matsushita M Tamiya T Lu YF Ichikawa T Date I Matsui H Ubiquitination-resistant p53 protein transduction therapy facilitates anti-cancer effect on the growth of human malignant glioma cells FEBS Lett 2005 579 3965 3969 10.1016/j.febslet.2005.06.021 15996664

[B42] Yin D Zhou H Kumagai T Liu G Ong JM Black KL Koeffler HP Proteasome inhibitor PS-341 causes cell growth arrest and apoptosis in human glioblastoma multiforme (GBM) Oncogene 2005 24 344 354 10.1038/sj.onc.1208225 15531918

[B43] Roth P Kissel M Herrmann C Eisele G Leban J Weller M Schmidt F SC68896, a novel small molecule proteasome inhibitor, exerts antiglioma activity in vitro and in vivo Clin Cancer Res 2009 15 6609 6618 10.1158/1078-0432.CCR-09-0548 19825946

[B44] Ge P Ji X Ding Y Wang X Fu S Meng F Jin X Ling F Luo Y Celastrol causes apoptosis and cell cycle arrest in rat glioma cells Neurol Res 2010 32 94 100 10.1179/016164109X12518779082273 19909582

[B45] Monticone M Biollo E Fabiano A Fabbi M Daga A Romeo F Maffei M Melotti A Giaretti W Corte G Castagnola P z-Leucinyl-leucinyl-norleucinal induces apoptosis of human glioblastoma tumor-initiating cells by proteasome inhibition and mitotic arrest response Mol Cancer Res 2009 7 1822 1834 10.1158/1541-7786.MCR-09-0225 19861404

[B46] Laurent N de Boüard S Guillamo JS Christov C Zini R Jouault H Andre P Lotteau V Peschanski M Effects of the proteasome inhibitor ritonavir on glioma growth in vitro and in vivo Mol Cancer Ther 2004 3 129 136 14985453

